# Leaving no one behind in prison: improving the health of people in prison as a key contributor to meeting the Sustainable Development Goals 2030

**DOI:** 10.1136/bmjgh-2020-004252

**Published:** 2021-03-26

**Authors:** Nasrul Ismail, Audrey Lazaris, Éamonn O'Moore, Emma Plugge, Sunita Stürup-Toft

**Affiliations:** 1 Centre for Public Health & Wellbeing, University of the West of England Bristol, Bristol, UK; 2 School for Policy Studies, University of Bristol, 8 Priory Road, Bristol BS8 1TZ, UK; 3 European Office for the Prevention and Control of Noncommunicable Diseases (NCD Office), World Health Organization, Moscow, Leontyevsky Pereulok, Russian Federation; 4 Health and Justice Team, Public Health England, London, UK; 5 School of Primary Care, Population Sciences and Medical Education, Faculty of Medicine, University of Southampton, Southampton SO16 6YD, UK; 6 Global Public Health, Public Health England, London, UK

**Keywords:** health policy, health systems, public health

## Abstract

Worldwide, approximately 11 million people are currently being held in prison, a number that has steadily grown since the turn of the 21st century. The prison population is more likely to suffer from physical and mental ailments both during and prior to their imprisonment due to poverty, social exclusion and chaotic lifestyles. Recognition of people in prison is noticeably absent from the Sustainable Development Goals (SDGs), despite the goals’ ethos of ‘leaving no one behind’.

We present the first analysis of how improving the health of people in prison can contribute to achieving 15 SDGs. Relevant indicators are proposed to fulfil these goals while meeting the existing international prison health standards. We also assess the political, economic and social challenges, alongside the unparalleled COVID-19 pandemic that can thwart the realisation of the SDGs. To reach the ‘furthest behind first’, prison health must be at the forefront of the SDGs.

**Summary box**

- The 11 million people in prison globally comprise the ‘left behind’, given their extensive health needs underpinned by adverse political, economic, environmental, social and lifestyle factors prior to imprisonment.
- As the Sustainable Development Goals (SDGs) pledge to leave no one behind, improving the health of imprisoned people can contribute to achieving 15 SDGs—from ending poverty in all forms to enabling peace, justice and strong institutions—while enabling states to simultaneously meet the existing international prison health standards.
- There are many opportunities for, and threats to, prison health in realising the attainment of SDGs that are institution specific (eg, overcrowding), as well as political (eg, populism) and economic conditions; the global COVID-19 pandemic has further magnified obstacles around the timely attainment of many goals.
- This analysis informs the development of standards and indicators in prisons that can subsequently be applied to wider areas of the criminal justice system and to different health services.

## Introduction

The Sustainable Development Goals (SDGs) 2030^1^ seek, among other aims, to catalyse transformations in global health. With 17 goals, 169 targets and 230 indicators that UN member states are responsible to deliver, measure and monitor, the SDGs are calibrated to be comprehensive and ambitious, pledging to ‘leave no one behind’ and requiring governments to reach ‘the furthest behind first’.^1^

Imprisoned people are undoubtedly the ‘left behind’. Numbering over 11 million globally at any one time, these people come from the most deprived sections of society and have extensive health needs which can be exacerbated by poor prison conditions.^2^ Compared with the general population, people in prison are more likely to suffer from physical and mental illnesses,^3 4^ due in large part to the political, economic, environmental, social and lifestyle factors prior to their imprisonment. The association between poverty and social exclusion and the negative impact of these factors on health is indisputable,^5^ and growing evidence demonstrates that health interventions on individuals in prison has positive impacts for their family and wider social network.^6^

Emerging evidence on the COVID-19 pandemic demonstrates that it is disproportionately affecting certain groups and widening health inequalities.^7^ It is therefore particularly important now, when the ‘left behind’ are at greater risk of becoming further behind, that we recognise that more can be done to improve the health and well-being of this vulnerable population.

Substantial progress has been made in translating the SDGs to areas such as mental health,^8^ maternal and adolescent health,^9^ and sexual and reproductive health.^10^ However, neither the Millennium Development Goals (MDGs), which preceded the SDGs, nor the SDGs themselves have made specific reference to the health of people in prison. This gap in international health strategies overlooks the potential contribution of prison health to the SDGs, with the result that imprisoned people become even further marginalised.

Therefore, this paper articulates the potential contributions of improved prison health to the attainment of the SDGs at a time when the global COVID-19 pandemic is threatening the timely attainment of many goals.^11^ In line with the WHO’s definition of ‘prisons’, we use the term to refer to institutions that hold people sentenced, on trial or awaiting sentence, to a period of imprisonment by the courts for offences against the law, as well as other forms of compulsory detention, such as police cells, immigration removal centres and secure mental health institutions, acknowledging that the term can vary from one country to another.^12^ We first propose a conceptual framework for a prison health agenda in 2030 that aligns with the SDGs. Second, we demonstrate how such a framework is compatible with existing international concordats. Although they are not legally binding, their political weight serves as a continual reminder to the government that it should protect individuals’ entitlement to health and healthy living provisions during imprisonment, which would demonstrate the government’s commitment to upholding the human right to healthcare. Finally, we carefully consider both the opportunities for and threats to prison health as these relate to the aspirations embedded in the SDGs to promote and sustain a real-world impact on policy and practice. This paper provides a first step towards developing a standard that can subsequently be applied to wider areas of the criminal justice system and different health services to enable monitoring across time and geographical regions.

## Intersections of the prison health agenda and the SDGs

**Figure 1:**
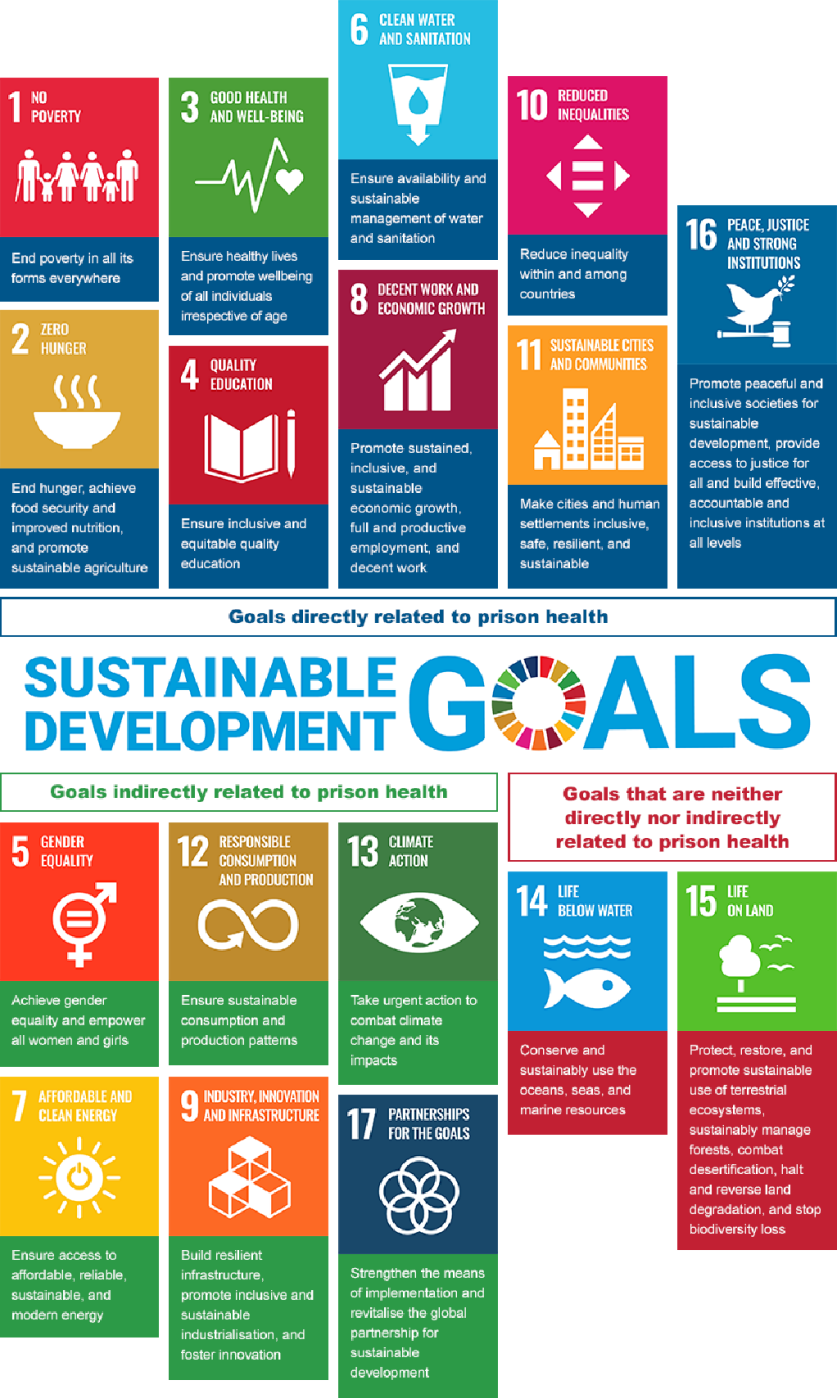
Conceptual framework of interactions between prison health and the SDGs. SDGs, Sustainable Development Goals.

The majority of SDGs are interrelated and mutually reinforcing (figure 1 and table 1). To ensure a holistic assessment, we adopt a broad lens on prison health, namely the WHO’s definition of health as ‘a state of complete physical, mental and social well-being and not merely the absence of disease and infirmity’.^13^ This characterisation requires us to consider the social determinants of health, which include poverty and education.

**Table 1:** Direct links of prison health to SDGs

SDGs	Potential contributions of prison health	Cross-over with existing international prison health policies
Goal 1—End poverty in all its forms everywhere	Considering that there is a bidirectional relationship between health and poverty, robust health services in prisons that are part of the general health services of the country similar to the UK, France and Norway^12^ will ensure the achievement of Targets 1.1 and 1.2 to eradicate extreme poverty and halve the no of those who currently live in poverty, respectively. It has been estimated that only 5% of people in prisons would be qualified to undertake higher education, partly because of a high prevalence of early school leavers among this cohort.^20^ As most people in prison will eventually be released into the community, prison education policies should prioritise a combination of basic skills (eg, upskilling IT skills of prisoners in Grundtvig, Denmark), vocational skills (eg, skilled worker intensive training, Facharbeiterintensivausbildung in Vienna, Austria) and higher education learning (eg, prison-university partnerships across the UK) to accrue a community dividend via improved future employability, reduced social costs of crime and successful reintegration into the community.^20^	The Mandela Rules 2015 The Bangkok Rules 2010
Goal 2—End hunger, achieve food security and improved nutrition and promote sustainable agriculture	A poor and insufficient diet can accelerate the probability of contracting a disease and hasten its progression. In order to provide access to safe, sufficient and nutritious food (Target 2.1), the issues of malnourishment (of both macronutrients and micronutrients) among prison populations, as well as the specific nutritional needs of female prisoners (including during pregnancy), children and young people in prisons must be addressed. Ensuring adequate nutrition to people in prisons, enabling healthy food choices and providing them with a basic understanding of the skills involved in day-to-day living, such as food preparation on a budget and healthier meal choices (which have been implemented in the USA, Western Europe and Canada) can contribute to the attainment of this goal.^21 22^	The Mandela Rules 2015 The Havana Rules 1990
Goal 3—Ensure healthy lives and promote well-being of all individuals irrespective of age	The following changes in areas of prison health can contribute to the achievement of Goal 3: Antenatal and postnatal care: Women in prisons often experience inadequate access to prenatal and postnatal care, alongside the prevalence of issues concerning maternal mortality and health complications from pregnancy and its termination.^23^ Enhanced perinatal care during detention, such as pregnancy-related services (eg, providing appropriate calories for meals and prenatal care) and accommodation (eg, special housing or a bottom bunk),^23^ can improve both short and long-term outcomes, help to reduce the global maternal mortality rate (Target 3.1) and eliminate the preventable deaths of newborns (Target 3.2). Communicable diseases: A combination of high-risk behaviours and inadequate access to preventive measures in prisons ensures the spread of communicable diseases.^24^ Prisons are a key setting in which to support the call to end the epidemics of AIDS, tuberculosis, malaria and hepatitis (Target 3.3). Access to voluntary and confidential testing, improved primary and specialist care services for diagnosis and treatment, prevention programmes (eg, needle and syringe programmes) and immunisation and vaccination will realise this aspiration.^24^ Non-communicable diseases: Alongside poor dietary intake and physical inactivity, cardiovascular issues have been reported to be the biggest killer in developed countries’ prisons.^4^ Prison health policies that combine prevention and treatment measures via physical activities, healthier diet, smoke-free prisons and both harm reduction and abstinence-based services on substance misuse^12 25^ can support the goal of reducing one-third of the incidents of premature mortality from non-communicable diseases (Target 3.4). Substance misuse: 90% of people in prison have substance misuse needs.^25^ Establishing a substance misuse strategy for prisons that includes both pharmacological and psychosocial interventions, such as opioid substitution therapy, harm reduction and drug-free unit strategies,^12^ can contribute to realising Target 3.5 and improve the prevention and treatment of substance misuse. Sexual and reproductive healthcare services: Women in prisons have a higher prevalence of sexually transmitted infections, such as chlamydia, gonorrhoea and HIV compared with men.^26^ Integrating sexual and reproductive health strategies and programmes within prisons, such as information and education, ensuring the availability of safer sex materials, contraception and sexual health clinics^12 26^ can help meet the aspirations of Target 3.7. Universal health coverage: People in prison should have access to universal healthcare that is safe, effective and affordable (Target 3.8). To achieve this target, prison healthcare provisions should be organised by the Ministry of Health, rather than by the Ministry of Justice or Interior.^12^ The notion of equivalent healthcare in prisons and the community has driven systemic improvement in the structure, organisation and regulation of healthcare in the UK, France and Norway that have adopted this arrangement.^12^ Tobacco control: The implementation of the WHO Framework Convention on Tobacco Control (Target 3·A) can be promoted by establishing smoke-free prisons, similar to England and Scotland.^27 28^ Considering that 80% of individuals in prison are smokers, compared with 24% of the general population, smoking bans and support for nicotine dependence will decrease tobacco use among individuals in prison and staff and improve air quality within prisons.^27 28^ Research and development of vaccines and medicines primarily for developing countries: To attain Target 3.B, prisons can accelerate vaccination opportunities to people who are often underserved by community vaccination programmes. For instance, experts have called for people in prisons to be prioritised for the national COVID-19 vaccination strategy, given their risk profile of underlying chronic conditions, age and living environment.^29^ Increase in health financing to improve the recruitment, retention and training of the health workforce, especially in developing countries: This goal can be promoted by the recruitment of an appropriate number of healthcare staff to work in prisons, as well as by ensuring that they undergo a process of professionalisation demonstrated to increase the quality of care in prisons, similar to the experiences of healthcare services in prisons in England.^30^ To support Target 3.D, health contingency plans should encompass prison establishments. For instance, to flatten the COVID-19 pandemic curve in prisons, plans should include risk communication, testing, social distancing, medical isolation or quarantine, operations guidance and the use of personal protective equipment.^7^ An examination of the dynamics of transmissions of COVID-19 in prisons, alongside infection control strategies and non-pharmacological interventions (eg, social distancing, population cohorting and isolation plans)^7^ could provide valuable insights into the risks and protective factors and help devise targeted interventions for COVID-19.	The Mandela Rules 2015 The Bangkok Rules 2010
Goal 4—Ensure inclusive and equitable quality education and promote lifelong learning opportunities for all	A large no of people who come in contact with prisons have been excluded from equitable, quality education and lifelong opportunities, which is a risk factor of offending.^31^ Encouraging and tailoring their participation in educational and vocational courses will enable them to participate more fully in educational opportunities,^31^ in line with Targets 4.1, 4.2 and 4.3. Meeting these targets will also eliminate gender disparities in education (Target 4.5), help achieve a high level of literacy and numeracy (Target 4.6), promote lifelong learning and contribute towards the nation’s economy. In emphasising Target 4.C, the quality of educational staff in prisons should be commensurable with that of educational staff in the community. Initiatives such as transnational learning from Portugal and the UK to support prison education staff in Romania, as well as training for new prison teachers in France conducted by both the Ministries of Education and Justice,^20^ can be considered.	The Mandela Rules 2015 The Bangkok Rules 2010 The Havana Rules 1990 The Beijing Rules 1985
Goal 6—Ensure availability and sustainable management of water and sanitation for all	Ensuring access to safe water (Target 6.1) and proper sanitation in prisons (Target 6.2) is key to meeting Goal 6. This need has been highlighted by the COVID-19 pandemic, where providing soap and tissue, using hand sanitiser, increasing laundry services and ensuring adequate personal protective equipment for staff are necessary infection control measures.^14^ Additionally, ending the use of unscreened toilets, providing regular access to showers and clean bedding, and measures to address water scarcity issues^12^ should be embedded as part of the resourcing policy of prisons (Target 6.4).	The Mandela Rules 2015 The Bangkok Rules 2010 The Havana Rules 1990
Goal 8—Promote sustained, inclusive and sustainable economic growth, full and productive employment, and decent work for all	Ensuring effective rehabilitation programmes in prisons will promote a healthier population that can better contribute to sustaining per capita economic growth (Target 8.1). Measures to be considered here include appropriate educational and vocational training programmes delivered in prisons^20^; encouraging enterprises to hire people with a history of imprisonment, particularly in those industries where workforce shortages are an issue^32^; and removing barriers to re-employment of people in prison, where appropriate (Target 8.3). Furthermore, considering that gaining employment after imprisonment can be challenging, productive employment post-incarceration can also be promoted via the provision of support to entrepreneurship^33^ (Target 8.5). Supporting the ability of people leaving prison to gain access to a bank account and financial advice (as part of their resettlement) can also facilitate their access to welfare programmes and their return to the workforce^32^ (Target 8.10).	The Mandela Rules 2015 The Bangkok Rules 2010 The Tokyo Rules 1990 The Havana Rules 1990 The Beijing Rules 1985
Goal 10—Reduce inequality within and among countries	As people in prison often come from the most deprived communities, incorporating them into the purview of Targets 10.1 and 10.2 can help sustain the income growth of the bottom 40% of the population, which can, in turn, reduce social inequalities and yield dividends in the form of safer, healthier and more resilient communities. Removing arbitrary and discriminatory practices that otherwise inhibit the rehabilitation of those who are imprisoned, such as stop and search,^34^ and reforming laws on drug use^35^ and sex work,^36^ is an important element in achieving this goal (Target 10.3). Given that economic conditions directly influence the availability of adequate healthcare for people in prison, another essential step is improving the regulation and monitoring of global financial markets^17^ (Target 10.5).	The Havana Rules 1990 The Beijing Rules 1985
Goal 11—Make cities and human settlements inclusive, safe, resilient and sustainable	In 115 countries, the data demonstrate that the number of people in prisons exceeds official prison capacities.^37^ Investments into the modernisation of prisons without necessarily increasing prison capacity (Target 11.1 on adequate housing), as well as into housing pathways for imprisoned people on release,^12^ will be vital to ensure that prisons are safe, resilient and sustainable by 2030. Although prisons are congregate settings by their very nature, clear policies for reducing communicable disease transmission, such as being able to physically distance people in prison to manage COVID-19, are critical.^7^ Other key measures include the restriction of prison transfers, enhanced medical appointments, regular and improved ventilation and disinfection and the establishment of protocols for isolation and quarantine that do not amount to solitary confinement conditions.^14^ Improving public transport for prisoners’ family members, especially those with children, and their friendship network,^38^ in line with Target 11.2 of providing affordable and sustainable transport by 2030, can also promote the rehabilitation of this population. Efforts to reduce the number of deaths of people affected by environmental disasters (Target 11.5) should take prisons into account at the contingency planning stage.^39^ ^40^ Creating green spaces in prisons (Target 11.7) can positively impact the mental well-being of people in prison and on the rehabilitative role of prison environments.^40^	The Mandela Rules 2015
Goal 16—Promote peaceful and inclusive societies for sustainable development, provide access to justice for all, and build effective, accountable and inclusive institutions at all levels	Targets 16.1 and 16.2 aspire to reduce all forms of violence and related death rates. Addressing the health-related drivers of violent behaviours and incidents (eg, substance misuse, mental illness and adverse childhood experiences)^12^ can reduce reoffending rates and create a safer community. Safety in prisons, which would benefit not only people in prison but also the prison staff, can be enhanced by adopting preventive mechanisms and improving reporting and response mechanisms for various forms of violence (eg, self-harm, arsons, riots, hostage-taking, bullying and victimisation).^12 17^ Prisons should aim to become more open, accountable and transparent about their investments, spending and service delivery^17^ (Target 16.6). Guaranteeing public access to information (Target 16.10) can propel prison institutions towards meeting this development goal. In compliance with Target 16.7 regarding ensuring participatory decision-making, as part of the inclusivity and empowerment drive, people in prison should be considered part of the process of developing and (where appropriate) delivering services in prisons. Several prisons in England have prison councils that allow prison representatives to consult on policy, practices and peer-support schemes.^41^ This strategy can be replicated elsewhere to improve inclusivity and empowerment.^41^	The Mandela Rules 2015 The Bangkok Rules 2010 The Tokyo Rules 1990 The Havana Rules 1990 The Beijing Rules 1985

SDGs, Sustainable Development Goals.

Adopting this broad lens on prison health reinforces the principle that good prison health is good public health. As prisoners often come from the most deprived sections of society and experience the greatest health needs,^5^ and the majority of prisoners will eventually be released into the community,^2^ prisons provide an opportunity to address those health needs, reduce inequalities and improve the population health overall. Meeting these goals will also reinforce the existing international policies and legal obligations outlined for prison health, detailed in table 2 and cross-referenced in our analysis of direct links of prison health to SDGs in table 1.

**Table 2:** Existing international prison health concordats

Existing international prison health concordats	Background information
United Nations Standard Minimum Rules for the Treatment of Prisoners 2015 (‘the Mandela Rules’)	These rules were first approved in 1957, and in 2015, they were revised and adopted as the Nelson Mandela Rules to honour the legacy of the late President of South Africa, who spent 27 years in prison in the course of his struggle, referred to above.^42^ These rules promote humane conditions of imprisonment, raise awareness about prisoners being a continuous part of society and value the work of prison staff as a social service of particular importance.^42^
United Nations Rules for the Treatment of Women Prisoners and Non-custodial Measures for Women Offenders 2010 (‘the Bangkok Rules’)	These rules were adopted by the UN General Assembly on 21 December 2010 to address the lack of focus on the needs of women in prisons, particularly with respect to healthcare, education and employment, and family contact.^43^
United Nations Standard Minimum Rules for Non-custodial Measures 1990 (‘the Tokyo Rules’)	Since 1990, these rules have urged the United Nations Member States to develop non-custodial measures within the legal system to reduce the rate of imprisonment. They range from using pretrial detention as a last resort to developing alternative sanction measures within the community by considering competing interests, such as human rights, social justice and rehabilitation.^44^
United Nations Standard Minimum Rules for the Administration of Juvenile Justice 1985 (‘the Beijing Rules’)	These 1985 rules seek to promote the health, well-being, and welfare of young people in prisons and assist them to lead a meaningful, crime-free life that fosters personal growth in the community.^45^
United Nations Rules for the Protection of Juveniles Deprived of their Liberty 1990 (‘the Havana Rules’)	These 1990 rules urge that the imprisonment of young people should be a last resort. To foster reintegration into society, juvenile institutions should attend to these young individuals’ health and welfare needs during detainment, with consideration for early release.^46^

## Indirect links of prison health to SDGs

Our assessment identifies six goals that indirectly link prison health to SDGs. To achieve gender equality and empower women and girls (goal 5), appropriate gender-specific health services for women in prison and viable alternatives to imprisonment for this population cohort can help reduce or put an end to discrimination against this gender (Target 5·1). Similarly, the development of trauma-informed services can help reduce the impact of violence on women, as well as increase their resilience (Target 5·2). In addition, goal 7 of ensuring access to affordable, reliable, sustainable and modern energy for all can be promoted by the appropriate use of clean and sustainable energy as part of prison infrastructure management. By building a resilient infrastructure and fostering innovation (goal 9), prison research networks such as the Worldwide Prison Health Research & Engagement Network can play a critical role in scrutinising the development and dissemination of prison health research thus enhancing scientific progress in the achievement of this goal by 2030 (Target 9·5).

The aims of goal 12 to ensure sustainable consumption and production, reduction of food waste through production and the supply chain (Targets 12·1 and 12·3), promotion of recycling and reuse (Target 12·5), achievement of sustainable production procurement and delivery of goods and services (Targets 12·6 and 12·7), and the improvement of education programmes regarding sustainable development, should be addressed with the prison population and environment in mind. Urgent action to combat climate change and its impacts via goal 13 should also include prisons as part of national and local disaster risk reduction and recovery plans, and the awareness of prison staff and inmates can be raised, as can their resilience or adaptive capacity to climate-related hazards and natural disasters (Targets 13·1 and 13·3). The COVID-19 pandemic has demonstrated the need to consider prisons in resilience planning.^14^ In realising goal 17 of revitalising the global partnership for sustainable development, the WHO Health in Prisons Programme and its associated initiatives can develop and enhance surveillance and data systems for prison health, particularly in low/middle-income countries, in order to meet Targets 17·9, 17·16, and 17·18. Doing so will improve cooperation and provide in-roads towards the establishment of a global prison health programme.

## Addressing the challenges to realising the SDGs through the prison health agenda

Opportunities for prison health to contribute towards SDGs come with unique challenges that are politically dependent and economically and socially contingent. Each government is mandated to incorporate the SDGs into its national planning and monitoring via the Voluntary National Reviews (United Nations, 2015).^1^ Action on the SDGs is therefore variable and sensitive to a wide range of political and socioeconomic factors. In addition, since December 2019, COVID-19 has presented an unparalleled global challenge with profound health, economic, social and political impacts. The response in prisons has provided a valuable moment of reflection on prison health issues.

Macroeconomic conditions are commensurable with a government’s commitment to providing sufficient financial resources for prison health. The price tag for delivering the SDGs is roughly between US$3·3 and US$4·5 trillion a year.^15^ The World Bank has predicted the worst recession for the global economy since World War II due to COVID-19,^16^ and in several European countries, fiscal austerity has persisted over the medium term. The resulting reduction in prison staff has been linked to an increase in violence, self-harm and suicide in prisons.^17^ As politicians are faced with competing fiscal demands, it is important to address the under-resourcing of health and welfare services in the criminal justice system and give proper consideration to prison health so that it can contribute to the SDGs.

A further challenge is posed by growing populism within countries, which might hinder progress in prison health that can contribute towards the SDGs. The trend towards tough stances on crime encourages a punitive approach to people in prisons, compromising the prison health agenda’s delivery.^17^ Given that widespread imprisonment is economically unsustainable and can in itself pose a public health risk both in prison and in local communities, as demonstrated by COVID-19, governments should consider alternatives for those who do not need to be confined for security and public protection purposes.^17^ While the regressive nature of the global pandemic exposes and adds to the current system’s vulnerability, countries across the globe, from the USA to Uganda and from Iran to Australia, have resorted to safe decarceration measures that go beyond secondary precautions, demonstrating how the pandemic presents us with an opportunity to accelerate the criminal justice reform that is already ongoing and critical to the attainment of SDGs.^14^ Progressive legislative changes and sentencing practices in a country can encourage alternatives to incarceration, emphasise that the deprivation of the liberty of individuals should only be the last resort, and reinforce the rehabilitation role in reducing the reoffending rate and contributing towards a safer and healthier society.^12^

In many countries, people in prisons have been side-lined within government responses to COVID-19 despite the fact that the potential for extensive outbreaks because of the higher prevalence of pre-existing poor health in imprisoned people and adverse environmental factors such as overcrowding and poor cleanliness and infection control, as well as the movement of staff and people in prison seeding and feeding outbreaks. In line with the WHO’s Moscow Declaration, prison health is public health. In countries where the risk of COVID-19 has been addressed in prisons, this has been successful in decreasing the infection risk to those imprisoned and to the wider community.^18^ An effective national public health response to COVID-19 includes prisons.

Continuity of care beyond prison is also critical to ensuring that the investment in health gains made in prison are continued in the community. Through the principle of equivalence in healthcare,^4^ prisoners should be entitled to receive access to healthcare of the same quality as anyone in the community. Since most people in prison spend the majority of their lives in the community, there is an opportunity to realise a community dividend by providing improved healthcare in prisons.^4^ Doing so can impact health service utilisation within the community on release, as well as reduce health inequalities. Prison and community health programmes should embrace close cooperation and operate cohesively to ensure that no one falls through the gap as people move from one setting to another.

Prison health is part of the wider domestic public health system, and its delivery requires a concerted effort across institutions if it is to make a significant contribution to the achievement of the SDGs. Additionally, involving the prison workforce and inmates themselves in delivering the SDGs at a microlevel of governance has the potential to ensure that action is carried out effectively at a local level and that it informs the national strategy. Including these groups in coproducing and codelivering the SDGs is a way to address the criticism regarding the undemocratic nature of the MDGs.^19^

Such a potentially large-scale operation inevitably prompts the question: ‘What would success in prison health look like in 2030?’ Considering the broad and far-reaching scope of the SDGs, adopting a robust monitoring scheme from the outset could be instrumental in holding the entire system accountable for producing results based on the agreed on indicators and outcomes. Future research could build on this recommendation by developing relevant indicators that could act as the cogwheels of the SDGs, as well as case studies which can be used to evaluate opportunities and celebrate successes in implementing the SDGs across different regions of the world. We are optimistic that our approach to considering the potential contribution to the SDGs of actions regarding a vulnerable population are transferable to other settings and populations, while remaining sensitive to different political and system landscapes.

## Conclusions

We have set out a novel conceptualisation of how prison health can contribute to meeting the SDGs by 2030 and have analysed how the prison health agenda can contribute to the attainment of aspirations for sustainable development even in a world challenged by a pandemic. While there are political, economic and social obstacles to delivering SDG ambitions, individual member states must seize the opportunity to effectively address these challenges at a national level through the work of policymakers, practitioners and academics.

Cooperation in transforming the vision of the SDGs into reality should benefit the development of all humans, including those who are currently disadvantaged, marginalised and excluded from global society. It is imperative that prison health be considered a useful and impactful action that can help attain the SDGs, reaching the ‘furthest behind first’ by ‘leaving no-one behind’.
